# Serum lipoprotein (a) and atherosclerotic changes in hemodialysis patients

**DOI:** 10.12861/jrip.2013.17

**Published:** 2013-06-01

**Authors:** Mahmoud Rafieian-Kopaei, Hamid Nasri

**Affiliations:** ^1^Medical Plants Research Center, Shahrekord University of Medical Sciences, Shahrekord, Iran; ^2^Department of Internal Medicine, Shahrekord University of Medical Sciences, Shahrekord, Iran

**Keywords:** Atherosclerosis, Diabetes mellitus, Hemodialysis, Intima-media thickness

## Abstract

**Introduction:** Lipoprotein (a) [Lp(a)] is considered as a risk factor for coronary atherosclerotic disorder and an increase in plasma Lp(a) concentration is usually seen in patients with kidney failure.

**Objectives:** We aimed in this study to evaluate the impact of plasma Lp(a) level on early changes of atherosclerotic vessels in stable hemodialysis (HD) patients.

**Patients and Methods:** In this clinical study 61 patients (50 non-diabetic (F=20 M=30) and 11 diabetic HD patients), receiving maintenance hemodialysis were included and serum Lp(a) was measured. B-mode ultrasonography of carotid intima-media thickness (cIMT) was also determined.

**Results:** The mean ±SD of patients Lp(a) was 58.5±19 mg/dL. The mean ±SD of diabetic patients’ Lp(a) was 62±12mg/dL and for nondiabetic ones was 57.7± 20mg/dL. In this study the thickening of Intima-media complex in diabetic group was more than non-diabetics, and no significant difference was found of Lp(a) between diabetic and non diabetic HD patients. There was a significant positive association between serum Lp(a) and cIMT.

**Conclusion:** Accelerated atherosclerosis seen in diabetic hemodialysis patients. Lp(a) might have an important role in progression of atherosclerosis to accelerate progressive atherosclerosis in these patients.

**Implication for health policy/practice/research/medical education:**

Accelerated atherosclerosis seen in diabetic hemodialysis patients. Lipoprotein (a) might have an important role in progression of atherosclerosis to accelerate progressive atherosclerosis in these patients.

## Introduction

Lipoprotein (a) is a macromolecule consisting a glycoprotein apolipoprotein (a) that is linked to apolipoprotein B-100 on LDL core (1).Its concentration is primarily determined genetically (2). Epidemiological studies have illustrated that, in high level, it is an independent risk factor for atherosclerotic coronary disorders (1,2). In renal failure, the plasma concentration of lipoprotein (a) [Lp(a)] may increase dramatically (1-3). However, the increase in Lp(a) level in these patients usually is not due to inherent .Kidneys might have an important role in Lp(a) metabolism (1-3). Indeed in beginning of chronic renal failure, Lp(a) starts to increase when glumerular filtration rate (GFR) reaches below 70 ml/min, (1-3), and dialysis procedure will be not able to reduce serum Lp(a) level . Irrespective of mechanisms which may be involved in increasing the Lp(a) level, this lipid might be a contributing factor in high frequency of atherosclerotic disorder observed in HD patients (1-3).

Atherosclerosis has been shown to be accompanied by some changes in arterial structure. Subtle structural changes such as arterial intima-media thickening (IMT) may occur early in the atherosclerotic disorder process (3,4). Using B-mode ultrasonography for evaluating early arteriosclerosis is noninvasive and safe to study superficial vascular districts, like carotid artery (3). Therefore, ultrasonic evaluation of carotid artery for intimae-media thickness may help identifying patients at risk for cardiovascular diseases (3). Carotid-IMT (cIMT) measurements are also strongly related to the extent of atherosclerosis in other vascular districts (3,4).

There is a significant association between conventional risk factors and increased arterial wall thickness (3,4), however, the effects of Lp(a) on IMT in hemodialysis patients is not fully understood.

## Objectives

In this study we, therefore, aimed to evaluate the effects of plasma Lp(a) level on early structural atherosclerotic vascular changes in a group of HD patients under maintenance hemodialysis.

## Patients and Methods

### Patients

This cross-sectional study was conducted on sixty-one patients on maintenance hemodialysis. Cigarette smoking, taking lowering drugs, recent MI, vascular diseases, body mass index (BMI) more than 25, as well as active or chronic infection were considered as exclusion criteria. Lp(a) was measured in patients by enzyme‏ immunoassay (ELISA) method using immuno-biological laboratories‏ (IBL) kits (Germany).

### Assessment of arterial intima-media thickening

Carotid sonography (Honda-Hs-2000 Sonograph with 7.5 MHZ linear probe) was done at the end of‏ diastolic phase by‏ a sonologist who was blind to history or lab data of‏ patients. For examination,‏ patients were placed in supine position with neck hyperextension and rotation of head to facilitate the performance of the procedure. The sites of measurements were at the‏ distal common carotid artery, first proximal internal carotid artery and area of bifurcation. IMT was measured at the plaque free areas (3,4). IMT was considered as the distance from leading edge of lumen-Intimae‏ interface of the far wall to the leading edge of the‏ media-adventitia interface of the far wall (3,4). IMT more than‏ 0‏.8‏ mm was considered abnormal (3-5).

### Ethical issues

(1) The research followed the tenets of the Declaration of Helsinki; (2) informed consent was obtained; (3) the research was approved by the institutional review board.

### Statistical analysis

For analysis we ‏ measured the mean of right and left‏ ‏ cIMTs. Data‏ were expressed as Mean±SD. Comparison between‏ groups was done using, Mann-Whitney U test, Kruskal Wallis test and Fisher's‏ exact test. To evaluate the correlations we used spearman`s rho test, partial correlation test with adjustment for age, also Phi‏ ‏ & Cramer's V test and Eta test were used. All statistical‏ analysis was performed using SPSS 11 (SPSS Inc., Chicago, IL, USA).‏ P of less than 0.05 was considered as significant.

## Results

The total patients were 61 (male=38 and female=23), consisting of 11‏ diabetic hemodialysis patients (F=3 and M=8) and 50‏ non diabetic hemodialysis patients (M=30 and F=20). Table 1  shows‏ the mean±SD of patients’ data. The mean age of the patients‏ was 46.5±16 years. The duration in which patients were on HD was 32±31 months. Serum‏ Lp(a) of patients was 58.5±19 mg/dL. Serum Lp(a) of diabetic and nondiabetic groups were 62±12 mg/dL and 57.7±20 mg/dL, respectively. The cIMT of diabetic‏ and non diabetic patients were 1.3±0.3 mm and 1±0.25mm, respectively. All HD patients were‏ hypertensive from stage one to stage three. In this study there was not any‏ significant difference for age or duration of hemodialysis treatment between male and female subjects‏) p>0.05). No significant difference for DM was also seen between two‏ sexes (p>0.05). There was not also significant difference for duration of‏ hemodialysis treatment or ages of the patients between‏ diabetic and non diabetic patients (p>0.05).‏ The difference between‏ diabetic and non diabetic groups was not significant for serum Lp(a), too (p>0.05). However, there was a significant difference for cIMT between‏ diabetic and non-diabetic patients (p<0.05). Statistical‏ analysis on cIMT with partial correlation test (after‏ adjustment for age) revealed no correlation between cIMT and duration of hemodialysis treatment‏ (p>0.05). Statistical analysis on Lp(a) showed a significant‏ positive correlation between cIMT and‏ lipoprotein(a) (r=0.330, p=0.008).

**Table 1:** cIMT and Lp (a) in patients.

	Total patients			Diabetic group			Non- Diabetic group		
	Mean±SD	Min	Max	Mean±SD	Min	Max	Mean±SD	Min	Max
Age (years)	46.5±16	15	78	57±16	27	78	47.8±16	15	78
Duration of hemodialysis (months)	32±31	2	108	22.6±22.4	3	60	34±33	2	108
cIMT (mm)	1.06±0.3	0.50	1.70	1.3±0.3	0.8	1.70	1±0.25	0.50	1.60
Lp(a) (mg/dL)	58.5±19	25	154	62±12	40	85	57.7±20	25	154

## Discussion

In this study there was more thickening of intima-media‏ complex in diabetic patients compared to non-diabetic ones. However, no significant‏ difference was found for lipoprotein (a) between diabetic and non-diabetic HD‏ patients. Pascasio‏ *et al*. observed a lot of vascular plaques in‏ patients suffering from Uremia. He concluded that the process of advance‏ atherosclerosis should be started with initiating of‏ renal failure. They suggested that HD treatment‏ might not be a potential factor for accelerating arthrosclerosis. In addition, they concluded that, progression of atherosclerosis‏ should be related to atherogenic factors which exist before‏ regular dialysis (5). To investigate, IMT of‏ 45 dialysis patients, Damjanovic and colleagues found higher mean cIMT in HD‏ patients compared to control group. They found a positive‏ association between cIMT and certain risk factors for‏ atherosclerosis (duration of dialysis, lipid parameters and age,) (6). The correlation of IMT and age of the patients or duration of hemodialysis in HD patients was investigated, by‏ Shoji and Hojs. In their studies, they did not find any relationship between IMT and‏ duration of HD (7-9). They concluded that hemodialysis patients might develop advanced atherosclerosis in the carotid arteries compared‏ with normal subjects (7-9). In agreement with our findings, in a study on 24‏ dialysis patients, Savage and colleagues noted the correlation between age and cIMT (10). Likewise, Kato *et al*. in a‏ study observed a significant‏ correlation between IMT and age on 219 hemodialysis patients (11).‏ Similarly, Baldassarre and colleagues in a‏ study on 100 patients, showed‏ higher values of cIMT in hypercholesterolemic‏ patients with plasma Lp(a) levels >30mg / dL than in those with lower levels. They concluded that elevated plasma levels of lipoprotein (a) might be considered as additional‏ independent factor associated with thickening of carotid‏ artery in subjects with severe hypercholesterolemia but‏ not in those with moderate hypercholesterolemia or‏ normal patients (12). Finally, Raitakari and colleagues in a study‏ on 241 healthy subjects found no association between‏ IMT and serum Lp(a) level, however, significant positive correlation could be seen between IMT and total‏ Cholesterol, LDL-C, LDL/HDL ratio, TG or age (13).

In the present study there was a significant positive association between serum Lp(a) and cIMT. Although, the extraordinary high mortality of HD patients are due to‏ cardiovascular disease, there are some interests toward nontraditional atherosclerotic cardiovascular diseases risk‏ factors such as Lp(a) which are high in HD patients . This needs more attention because of its effects on acceleration of rapid progressive atherosclerosis seen in HD patients.

## Conclusion

Diabetic HD patients have more accelerated atherosclerosis. Lp(a) as‏ a nontraditional factor in progression of atherosclerosis can have a more important role in acceleration of rapid progressive‏ atherosclerosis observed in these patients.‏

## Acknowledgments

We would like to thank research deputy of Shahrekord University of Medical Sciences for financial support and Dr. M. Mowlaei (sonologist) for carotid ultrasonographies.

## Authors' contributions

HN designed and performed the research. MRK wrote some parts of paper. MRK edited the draft. HN prepared the final draft.

## Conflict of interests

The authors declared no competing interests.

## Ethical considerations

Ethical issues (including plagiarism, misconduct, data fabrication, falsification, double publication or submission, redundancy) have been completely observed by the authors.

## Funding/Support

None.
